# Association between socioeconomic status and mental health among China’s migrant workers: A moderated mediation model

**DOI:** 10.1371/journal.pone.0274669

**Published:** 2022-09-15

**Authors:** Yubo Shao, Huang Ying, Xiaoming Li, Lian Tong

**Affiliations:** 1 Key Laboratory of Public Health Safety, School of Public Health, Fudan University, Ministry of Education, Shanghai, China; 2 Education and Behavior, University of South Carolina, Columbia, SC, United States of America; PLOS ONE, UNITED KINGDOM

## Abstract

Mental health problems are prevalent among China’s internal migrant workers. However, research on the relationship between socioeconomic status (SES) and mental health is insufficient. Therefore, this study, utilizing the China’s National Dynamic Monitoring Survey data from a sample of 15,997 migrant workers aged 15–59 years to explore differences in the relationship between migrants’ objective and subjective SES and mental health status in 2015. Both the mediating effect of perceived interpersonal discrimination and the moderating effect of age were examined through structural equation modeling. The results indicated that subjective SES has a stronger direct relationship with mental health than objective SES. Perceived interpersonal discrimination mediated the association between subjective SES and mental health. Furthermore, a much stronger relationship was found between subjective SES and perceived interpersonal discrimination among migrants older than 24 years of age than younger migrant groups. The results showed that, compared with traditional objective SES indicators, subjective SES could be a more sensitive index for identifying those migrant workers with a high risk of mental health problems. In addition, reducing interpersonal discrimination toward migrants can alleviate their mental health problems. And we should pay more attention to older migrant workers since they are more likely to prone to interpersonal discrimination and mental health issues.

## Introduction

As the most populated country, China also has the largest internal migrant population in the world [1]. According to the most recent figures released by the National Health and Family Planning Commission of PRC, there were about 244 million internal migrants in China in 2017, accounting for 17.6% of the total population [2]. Migrant were defined as those who had resided in a location that is different from their place of household registration [1]. Migrant workers were drawn by a wide array of job opportunities in economically developed areas in the east. While migrant workers have contributed greatly to China’s urban economic growth and development, they have not seen corresponding improvements in their socioeconomic status (SES). Thus, various forms of inequality have emerged between migrants and urban residents [3]. Previous studies have found a clear evidence of disparities in wages, social security, and employment opportunity between local and migrant workers [3,4]. Moreover, many migrant workers still live in crowded, substandard housing conditions [5,6], and engage in unsanitary, difficult, and dangerous work [1]. Despite various efforts to improve migrant workers’ status, they are still considered as one of China’s most disadvantaged social groups [7].

A number of previous research suggested that the socioeconomic status (SES) is significantly associated with mental health problems among migrants in China [8]. However, some knowledge gaps exist. For example, most of the research has used some objective measures of SES (e.g., education attainment, income, occupational prestige) which may not be sensitive to the migrant populations due to their life context [9–11]. In addition, few researches have taken into consideration the potential confounders that may mediate or moderate the relationship between SES and mental health among migrants.

The objective measures of SES [12], while commonly used in the literature, are not only subject to reporting bias but also less sensitive to the migrant population as they are generally low-educated with low income. In contrast, some research has used subjective measures (e.g., people’s relative SES compared with others) [13] and found significant association between subjective SES and mental health issues among migrant workers [8,14]. Although objective SES provides the material foundation for subjective SES, there is no high correlation between the two indicators [15]. This may be because that subjective SES is affected by people’s perceptions of their relative position within certain well-defined groups. Various studies conducted in Western contexts have reported different effects of objective and subjective SES on health outcomes with subjective SES being more strongly related to mental health and well-being than objective SES [16]. One study found that subjective SES could better predict a decline in health status over time [17]. However, it remains unclear whether the two types of SES (objective and subjective) are associated at different magnitudes with the mental health problems of Chinese migrant workers. Hence, the research hypothesis for this study are list as follows:

***Hypothesis 1a*.** *Good objective SES will positively affect the mental health status directly*.
***Hypothesis 1b*.** *Good subjective SES will positively affect the mental health status directly*.
***Hypothesis 1c*.** *Subjective SES has a stronger association with mental health than objective SES*.

In addition, some salient social factors (e.g., discrimination) and demographic factors (e.g., age) may play significant roles in migrants’ mental health status. As a socially marginalized group, migrant workers often face interpersonal discrimination in urban destinations. Perceived interpersonal discrimination is the subjective feeling of being unequally treated by other people; such biased treatment can assume verbal, nonverbal, or paraverbal forms, such as avoiding eye contact, being standoffish, minimizing interaction, or showing overt hostility [18]. For example, some Chinese migrants who have lived and worked in a city for years are treated as “outsiders” [19], and are even perceived as a threat to the society [5]. Studies have found that people with lower SES are generally treated negatively and are more likely to perceive higher levels of discrimination. For instance, a study of 3,082 adults based on the Midlife in the United States survey found that some people’s perceptions of being discriminated against could be attributable to their low SES [20]. Similarly, another study verified that poverty can result in higher levels of perceived discrimination among adolescents [21]. Meanwhile, perceived interpersonal discrimination has been widely found to increase the possibility of negative mental health outcomes. Specifically, people with higher levels of perceived interpersonal discrimination have been found to be much more likely to report anxiety [9,22], depression [22–24], sadness [23], and low self-esteem [25], among other mental health problems. Thus, it is hypothesized:

***Hypothesis 2***: *Perceived interpersonal discrimination mediates the relationship between SES and mental health*.

**Fig 1:**
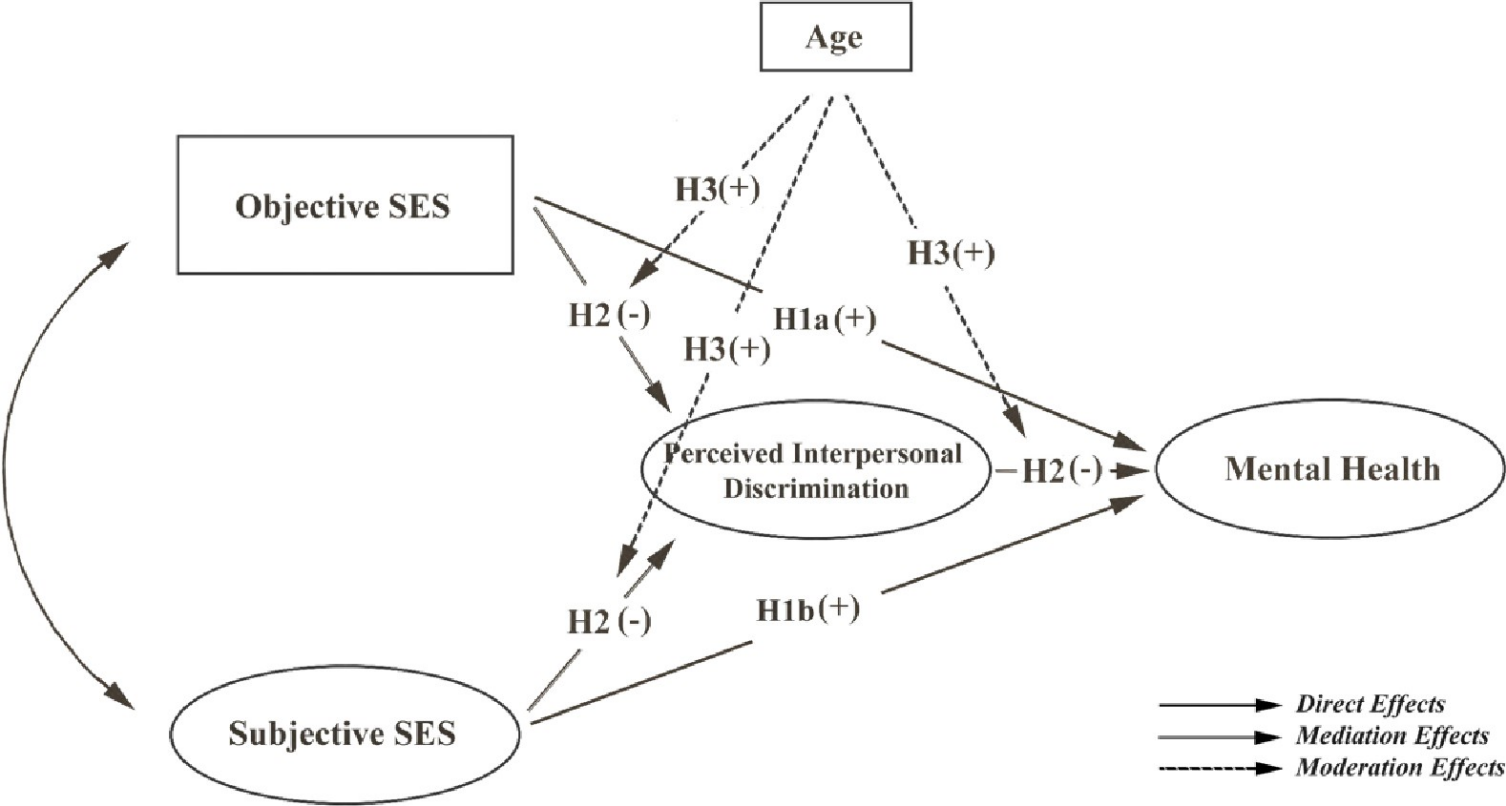
Conceptual framework. **Note.** SES: Socioeconomic status; H1a (+): Hypothesis 1a, which is expected to be positive.

Moreover, the effects of SES and discrimination on mental health may also differ by age group. The main motives for migration may be different among different age groups. While the older migrants may choose coming to the cities to escape poverty, the younger migrants are often attracted to urban living and choose coming to the cities to seek a new lifestyle [1]. The younger migrant workers are typically better educated, have higher ambitions, and tend to be a part of urban society [26,27]. Further, the perceived gaps in SES between them and others might have to do with their older colleagues in the workplace or older family members rather than their peers [28,29]. Younger people can move out of their low SES group as they grow older, and the likelihood of upward mobility can reduce perceptions of discrimination [30]. Young migrant workers are also generally more optimistic than the older ones [31], which could lead them to perceive less interpersonal discrimination despite their low SES. For older people, however, upward mobility can be difficult if they have attained a stable job; therefore, being stuck in a low SES, they might perceive higher interpersonal discrimination. In addition, according to Carstensen’ s socioemotional selectivity theory, people become increasingly fastidious in seeking emotional support from others as they age [32]. As such, when older people feel discriminated against, they may receive emotional support from a more limited range of people than younger people do and therefore experience more mental health problems. Studies have also found that older adults tend to use more passive strategies, such as suppression, avoidance, and rumination, when they regulate their emotions [33–35]; such actions may have detrimental effects on mental health [36,37]. Thus, older migrants will be more likely to face mental health problems than younger migrants when they perceive discrimination (Fig 1). Hence, it is hypothesized:

***Hypothesis 3***: *Age moderates the strength of the indirect effect of SES on mental health via perceived interpersonal discrimination*, *and the indirect effect is stronger for older migrants*.

To address the knowledge gaps in research on the relationship between SES and mental health among migrants, this study was designed to compare the association of objective and subjective SES with mental health among China’s migrant population. We also examined the mediating effect of perceived interpersonal discrimination to identify the specific mechanism in the association between SES and mental health as well as the role of age in moderating the relationship between SES and mental health.

## Method

### Data source

The data used in this study was a secondary data source, which collected originally from the National Dynamic Monitoring Survey of Social Integration and Mental Health in the Chinese Floating Population by the National Health and Family Planning Commission (NHFPC) of China in 2015. The raw data was acquired through the website of National Health and Family Planning Commission of China (www.nhfpc.gov.cn), then the raw data were cleaned and analyzed by authors.

### Participants

The participants in this survey were sampled from prefecture-level cities or districts located in eight Chinese provinces (one city/district per province). The participants were internal migrants aged 15–59 years in the recruitment time. Migrants were defined as those who had lived in a local residence over one month without a local household registration (“hukou”) at the time of the survey [1]. University students are excluded from the migrated population in this study. The participants were recruited using stratified, multistage probability proportional to size (PPS) sampling method. In the first stage of sampling, townships (or streets) were randomly selected using the PPS method, followed by sampling of villages or neighborhoods in the second stage. In each village or neighborhood, 20 individuals from different households were randomly selected in the third stage. A total of 2,000 migrants were selected from each of the eight prefecture-level cities (or districts). If the selected respondents could not be visited during the survey, they were replaced by other individuals based on the principle of “same sex, similar age, and a similar length of time living in the receiving area.” Due to missing data on key study variables, three cases were excluded, resulting a final sample size of 15,997 in the current study.

### Ethical approval and consent to participate

Data from the National Internal Migrant Dynamic Monitoring Survey are available to researchers who have received permission from the National Population and Family Planning Commission. Written informed consents were obtained from all participants. The analysis of public access data was exempted by the local IRB of Medical ethics committee of School of Public Health of Fudan University. All data were provided in an anonymized format.

### Measures

#### Objective socioeconomic status

This study assessed objective SES based on monthly household income, which is the most commonly used and most reliable indicator of objective SES [38,39]. Each respondent was asked to report their monthly household income in Chinese currency Yuan (CNY). We minimized the effect of cross-city economic disparities by subtracting the minimum monthly wage decreed by the Ministry of Human Resources and Social Security of China. This adjusted monthly income was divided into five levels using quantile classification. The cutoff points were 1675 CNY, 3192 CNY, 4550 CNY, and 6530 CNY (the exchange rate was approximately $1 USD = 6.14 CNY at the time of survey). People in the level 1 group had the lowest household income, while those in the level 5 group had the highest.

#### Subjective socioeconomic status

Migrant workers’ subjective SES was evaluated by the MacArthur scale [40], which has been used for Chinese rural-to-urban migrant research previously [39,41]. Each respondent was shown a picture of a ladder with ten rungs, described as follows: “Think of this ladder as representing where people stand in our society. At the top of the ladder are the people who are the best off—those who have the most money and the best jobs. At the bottom are the people who are the worst off—those who have the least money and worst jobs.” Respondents were asked to report where they saw themselves on this ladder compared with three groups of people: (1) their family members, friends, and co-workers in their hometown; (2) their family members, friends, and colleagues at their current residence; and (3) people in the large society. A higher position in the ladder represents a perception of a relatively higher standing in the socioeconomic hierarchy in comparison with others. The internal consistency estimates (Cronbach’s alpha) were 0.856 for the three subjective SES ratings in this study.

#### Perceived interpersonal discrimination

Perceived interpersonal discrimination was measured based on the following four items: (1) “I feel the local residents are not willing to see me as one of them”, (2) “I feel the local people are not willing to be neighbors with me”, (3) “I feel the local people don’t like me”, and (4) “I feel the local people despise me”. Items were rated on a four-point Likert scale ranging from 1 (strongly disagree) to 4 (strongly agree). A higher score in each item indicated a higher level of perceived interpersonal discrimination. The Cronbach’s alpha was 0.903 for the current sample.

### Mental health

Mental health status was measured using the K6 scale [42], which has been proven effective for screening psychological distress symptoms among adults [43–45] and youth [46–48]. K6 is a six-item scale that asks respondents how often they had felt each the following symptoms in the past 30 days along a five-point scale ranging from 1 = all of the time to 5 = never): (1) nervous, (2) hopeless, (3) restless or fidgety, (4) so depressed that nothing can cheer you up, (5) everything is an effort, and (6) worthless. With appropriate recoding, the sum of the scores for the six items was used as a composite score ranging from 6 to 30. A higher composite score indicates a lower level of mental health problems. The Cronbach’s alpha was 0.817 for the sample in the current study.

### Age

Information on each participant’s age was collected during the survey. For the purpose of data analysis in the current study, age was used as a dichotomous variable using the United Nations’ definition of “youth” with those 24 years of age or younger being the “youth group”, and the rest (>24) being the “adult group” [49].

### Statistical analysis

Descriptive analysis was conducted using R version 3.6.0. [50] Structural equation modeling (SEM) was performed in AMOS 24.0 to examine the hypothesized relationships among the key study variables. Considering the cultural adaptability of the main rating scales used in this study, we first performed confirmatory factor analysis (CFA) to ensure the unidimensionality of each latent structure. Items with standardized factor loadings smaller than 0.6 were deleted [51]. Then, the composite reliability (CR), convergent validity, and discriminant validity of each latent construct were assessed. The method of SEM is a good method to explore the mediating effect, which is consistent with objectives of this study. SEM is also a good method to explore the causal relationship for cross-sectional data. The SEM analysis was based on the covariance matrix and performed using Browne’ s asymptotically distribution-free estimation method (ADF) [52]. The ADF method was suitable for the current data type, since the variables used in SEM were ordering categorical variable and continuous variables with non-normal distribution. Moreover, the substantial sample size was also a strength for this analysis. The standardized path coefficients between latent variables were computed. We calculated the values of *β* statistic for testing the significance of association. The bias-corrected bootstrapping with 5,000 bootstrapped samples were used to assess the confidence interval of the standardized path coefficients, the comparison of the direct effect of objective and subjective SES on mental health, and the test for the mediating role of perceived interpersonal discrimination [53,54]. We also used bias-corrected bootstrapping with 5,000 bootstrapped samples to test whether the mediation effects were contingent on age [53]. Model performance was evaluated by multiple goodness-of-fit indexes, and good fits were indicated by the comparative fit index (CFI), >0.9; goodness-of-fit index (GFI), >0.9; adjusted goodness-of-fit index (AGFI), >0.9; root-mean-square error of approximation (RMSEA), <0.08; and standardized root-mean-square residual (SRMR), <0.08 [55–57].

## Results

### Sample characteristics

The mean age of the migrants in this study was 32.69 years, with a standard deviation of 8.72. About 18.3% were youth (≤24 years of age), and 55% were men (Table 1). The majority of respondents were married (74.6%). Regarding education, 85.2% had finished no more than postsecondary school, and among them, 59.9% had finished no more than junior high school.

**Table 1:** Demographic characteristics and mental health status of the study sample (N = 15,997).

Variables	N (%)	Total score of K6 scale (mean, SD)
***Entire sample***	15,997 (100%)	26.58 (3.07)
***Age (mean*, *SD)***	32.69 (8.72)	
≤24	2,924 (18.3%)	26.38 (3.10)
>24	13,073 (81.7%)	26.61 (3.06)
***Gender***		
Male	8,798 (55.0%)	26.60 (3.11)
Female	7,199 (45.0%)	26.55 (3.02)
***Ethnicity***		
Han	15,434 (96.5%)	26.57 (3.08)
Minority	563 (3.5%)	26.71 (2.83)
***Marital Status***		
Married	11,941 (74.6%)	26.70 (3.05)
Unmarried	4,056 (25.4%)	26.22 (3.11)
***Education***		
≤ Junior high school	9,588 (59.9%)	26.71 (2.99)
Senior high school	4,051 (25.3%)	26.40 (3.18)
≥ Postsecondary	2,358 (14.8%)	26.34 (3.19)
***Household Monthly Income (CNY)***		
≤3,000	3,306 (20.7%)	26.21 (3.16)
3,000–6,000	7,203 (45.0%)	26.54 (3.08)
6,000–9,000	3,386 (21.2%)	26.79 (2.95)
≥9,000	2,102 (13.1%)	26.91 (3.00)
***Hukou***		
Agricultural	13,757 (86.0%)	26.60 (3.03)
Nonagricultural	2,240 (14.0%)	26.42 (3.30)
***Range of Migration***		
Cross province	8,769 (54.8%)	26.53 (3.15)
Within province	7,228 (45.2%)	26.63 (2.98)

### Confirmatory factor analysis

In the CFA, we removed the item “I feel the local people are not willing to accept me as one of them” from the subscale of “perceived interpersonal discrimination” and the item “worthless” from the mental health subscale since their factor loadings were smaller than 0.6. Table 2 shows the CFA results for the final measurement models. All the standardized factor loadings of the remaining items were greater than 0.6. All the latent constructs had high internal consistency, with CR ranging from 0.817 to 0.903. The three latent constructs showed an acceptable convergent validity with the average variance extracted (AVE) being 0.682, 0.766, and 0.486, respectively. Also, the correlation coefficients between subjective SES and interpersonal discrimination, subjective SES and mental health, and interpersonal discrimination and mental health were -0.118, 0.197, and -0.176, respectively. The absolute value of these inter-construct correlations was smaller than the square root of AVE (0.826 for subjective SES, 0.875 for perceived interpersonal discrimination, and 0.697 for mental health), which suggested an acceptable discriminant validity [58].

**Table 2:** Measurement models of key study variables.

Construct	Indicators	Unstd.	SE	*t*-value	Std.	CR	AVE
***Subjective*** ***SES***	S1: Compared with hometown	1.000			.824	.864	.682
	S2: Compared with current residence	1.121	.011	97.648	.942		
	S3: Compared with the whole society	.854	.011	79.874	.692		
***Perceived*** ***Interpersonal*** ***Discrimination***	P1: Local people are not willing to be neighbors with me	1.000			.796	.907	.766
	P2: Local people dislike me	1.120	.010	106.943	.954		
	P3: Local people despise me	1.042	.010	99.757	.868		
***Mental Health***	M1: Nervous	1.000			.692	.825	.486
	M2: Hopeless	.698	.014	51.052	.644		
	M3: Restless or fidgety	.973	.014	69.290	.734		
	M4: Depressed	.981	.015	67.106	.742		
	M5: Everything was an effort	.873	.014	61.066	.669		

**Note.** SES: Socioeconomic status; Unstd.: Unstandardized factor loading; SE: Standard error; Std.: Standardized factor loading; CR: Composite reliability; AVE: Average variance extracted.

### Structural equation modeling

**Fig 2:**
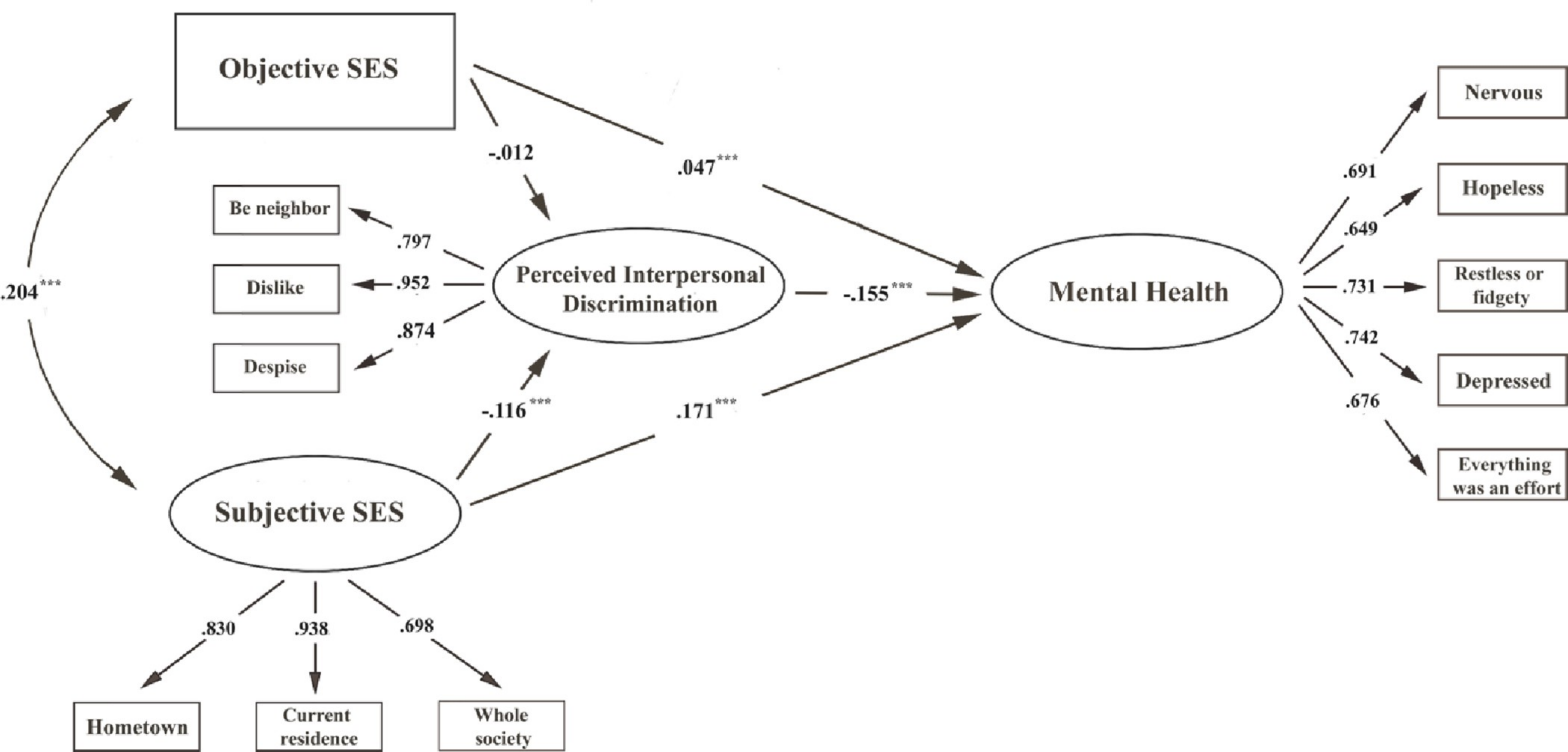
Association between socioeconomic status and mental health among Chinese migrants (structural equation model). **Note.** Standardized factor loadings and path coefficients are shown; ***p < 0.001. Model fits: GFI = .987, AGFI = .979, CFI = .949, RMSEA = .028, and SRMR = 0.018.

Table 3 shows the descriptive statistics, bivariate correlations of measurement indicators, and Cronbach’s *α* for the latent constructs. All indicators were significantly correlated with each other (*p*<0.001, *two*−*tailed*). SEM analysis confirmed that the hypothesized model fits the data well (GFI = 0.987, AGFI = 0.979, CFI = 0.949, RMSEA = 0.028, and SRMR = 0.018). Furthermore, all the standardized factor loadings were larger than 0.6, suggesting that each item was a good measurement indicator of the desired latent construct (Fig 2). Correlation analysis showed that the mental health had significantly positive correlations with objective SES, as well as subjective SES in each subscale (see Table 3).

**Table 3:** Descriptive statistics and correlation coefficients of the indicator variables (N = 15,997).

	1	2	3	4	5	6	7	8	9	10	11	12
***Objective SES***	1.000											
***Subjective SES***			**(.856)**									
Compared with hometown	.175***	1.000										
Compared with current residence	.181***	.776***	1.000									
Compared with the whole society	.159***	.570***	.651***	1.000								
***Perceived Interpersonal Discrimination***						**(.903)**						
Local people are not willing to be neighbors with me	-.034***	-.090***	-.089***	-.065***	1.000							
Local people dislike me	-.035***	-.095***	-.094***	-.060***	.760***	1.000						
Local people despise me	-.034***	-.102***	-.110***	-.082***	.691***	.828***	1.000					
***Mental Health***											**(.817)**	
Nervous	.031***	.115***	.122***	.128***	-.088***	-.088***	-.086***	1.000				
Hopeless	.065***	.112***	.107***	.079***	-.119***	-.136***	-.133***	.463***	1.000			
Restless or fidgety	.066***	.114***	.128***	.127***	-.090***	-.095***	-.097***	.549***	.437***	1.000		
Depressed	.069***	.110***	.118***	.105***	-.116***	-.130***	-.122***	.452***	.482***	.529***	1.000	
Everything was an effort	.081***	.120***	.132***	.118***	-.117***	-.128***	-.118***	.427***	.425***	.456***	.536***	1.000
***Descriptive Statistics***												
Mean	2.983	5.789	5.472	4.639	1.925	1.841	1.810	4.185	4.729	4.207	4.399	4.353
SD	1.414	1.634	1.603	1.663	.711	.664	.679	.781	.588	.715	.715	.705

**Note.** Figures in parentheses are Cronbach’s α of the latent construct. SES: Socioeconomic status.

***p < 0.001.

### Associations between SES and mental health

The SEM results indicated that both objective SES (*β* = 0.047; *p*<0.001, *two*−*tailed*) and subjective SES (*β* = 0.171; *p*<0.001, *two*−*tailed*) were directly associated with mental health. Specifically, higher objective and subjective SES predicted less mental health problems. Moreover, the bootstrapping results showed that subjective SES was more strongly associated with mental health than objective SES (see Table 4).

**Table 4:** Unstandardized direct, indirect, and total effects of the structural equation model.

Effect	Point Estimate		Bias-Corrected 99% CI	
			Lower	Upper
***Path*: *Objective SES → Mental Health***				
** Direct effect**		.018***	.009	.027
** Indirect effect**		.000	-.001	.002
** Total effect**		.018***	.010	.027
***Path*: *Subjective SES → Mental Health***				
** Direct effect**		.078***	.067	.091
** Indirect effect**		.008***	.006	.011
** Total effect**		.086***	.074	.099
***Difference in direct effect***				
		-.060***	-.077	-.044

**Note.** Significance tests were based on bias-corrected confidence intervals derived from 5,000 bootstrapped samples.

***p<0.001.

### Mediating effect of discrimination

As shown in Table 4, subjective SES was a protective factor for perceived interpersonal discrimination (*β* = −0.116; *p*<0.001, *two*−*tailed*), and a low level of perceived interpersonal discrimination in turn resulted in fewer mental health problems (*β* = −0.155; *p*<0.001, *two*−*tailed*). Objective SES was negatively associated with perceived interpersonal discrimination (*β* = −0.012), but the magnitude did not reach statistical significance at *α* = 0.05 (*p* = 0.16, *two*−*tailed*). Bootstrapping confirmed the existence of the mediating role of perceived interpersonal discrimination between subjective SES and mental health (bootstrapping 99% CI = [0.006, 0.011]). However, there was insufficient statistical evidence to support the role of interpersonal discrimination in mediating the direct effect of objective SES on mental health (bootstrapping 99% CI = [-0.001, 0.002]).

### Moderating effect of age on mediation model

Table 5 shows the moderation analysis results with unstandardized coefficients. Age moderated the mediation model of *subjective SES*→*perceived interpersonal discrimination*→ *mental health*. For people 24 years of age or younger, the association between subjective SES and discrimination (first-stage simple effects of the mediated relationship) (*β* = −0.037; p<0.001, *two*−*tailed*) and the association between discrimination and mental health (second-stage simple effects of the mediated relationship) (*β* = −0.118; *p*<0.001, *two*−*tailed*) were both significant, resulting in a significant indirect effect (*β* = 0.004; *p*<0.001, *two*−*tailed*). For those over 24 years of age, the first-stage (*β* = −0.062; *p*<0.001, *two*−*tailed*) and second-stage (*β* = −0.153; *p*<0.001, *two*−*tailed*) simple effects of the mediated relationship were both significant as well, resulting in a significant indirect effect (*β* = 0.009; *p*<0.001, *two*−*tailed*). Moreover, the indirect effect was significantly stronger in the old-age group than in the young-age group (Δ*β* = −0.005; *p* = 0.027, *two*−*tailed*); however, the second-stage simple effect did not significantly differ between the two age groups (Δ*β* = −0.035; *p* = 0.18, *two*−*tailed*).

**Table 5:** Moderated mediation results across age groups.

Moderate variable level	Subjective SES → Perceived Interpersonal Discrimination → Mental Health						
	Stage					Indirect effects *(P*_*S→P*_ *×P*_*P→M*_*)*	
	First stage *(P*_*S→P*_*)*		Second stage *(P*_*P→M*_*)*				
***Age***_***(young)***_ ***(≤24)***		-.037***		-.118***			.004***
***Age***_***(old)***_ ***(>24)***		-.062***		-.153***			.009***
***Difference between age***_***(young)***_ ***and age***_***(old)***_		-.024*		-.035			.005*

**Note.** SES: Socioeconomic status. P_S→P_: Path “Subjective SES → Perceived Interpersonal Discrimination”; P_P→M_: Path “Perceived Interpersonal Discrimination → Mental Health.” Significance tests were based on bias-corrected confidence intervals derived from 5,000 bootstrapped samples.

*p < 0.05;

**p < 0.01;

***p < 0.001.

## Discussion

Mental illness is a global problem that places a great burden on both the individuals and society [59,60]. This study enriches the literature by providing three new findings: First, subjective SES was more strongly related to mental health status than objective SES among Chinese migrant workers. Second, perceived interpersonal discrimination mediated the direct relationship between subjective SES and mental health but did not mediate the direct relationship between objective SES and mental health. Third, age moderated the indirect relationship between subjective SES and mental health via perceived interpersonal discrimination, and this mediated relationship was stronger for older people.

The finding regarding the stronger predictability of subjective SES than objective SES can be partly explained by the fact that the value of SES essentially depends on how people perceive them; that is, the things some groups care about might not be highly valued by others [61]. People with a higher subjective SES usually have fewer SES-related psychological stresses, resulting in less mental health problems [62,63]. Chinese migrants are a diverse group consisting of people with different educational and occupational backgrounds [64] as well as different migration motives and goals [65]. Therefore, objective SES, a measurement that is often quantified without considering the migrants’ life circumstance and environment, might not accurately predict the perceived stresses that can produce mental illness. The explanation might also lie in the theory of social comparison processes [66]. The theory depicts that people’s “happiness,” which is considered as a key aspect of mental health [67], depends on comparisons with surrounding population rather than with distant ones. In China, class differences are also found within migrant groups. This study also found that perceived interpersonal discrimination was a mediating factor in the relationship between subjective SES and mental health problems. Consistent with previous studies [9], subjective SES showed a significant link with perceived interpersonal discrimination among migrant workers. Moreover, perceived interpersonal discrimination was closely related to mental health problems, partly because discrimination by local residents can be considered a chronic social-environmental stressor [21]. However, this study found that objective SES was no longer related to perceived interpersonal discrimination after taking subjective SES into consideration. According to the theory of relative deprivation [68], people will feel they are unfairly treated when they compare themselves to some reference standard. Studies have documented large SES gaps between urban and migrant workers engaged in the similar type of work with the same performance levels [69,70]. This is viewed as a form of interpersonal discrimination by migrant workers, regardless of whether they have a high or low objective SES among migrant groups.

This study also suggested the moderating role of age in the relationship between subjective SES and mental health. Specifically, the link between subjective SES and perceived interpersonal discrimination was stronger among older migrants, resulting in a stronger indirect relationship between subjective SES and mental health. Chinese internal migrant workers generally perform “cheap labor” when they are still young [71]. Their wages tend to peak early and decline over time, along with the decline in physical strength [72]. Thus, since young people can potentially change their SES in the future, the prospect of upward mobility may reduce their perceptions of interpersonal discrimination [30]. However, opportunities for advancement generally diminish with age, which can give rise to an increased sense of discrimination.

The findings in this study have several important implications for promoting the mental health of migrant workers. First, subjective SES is a more sensitive index for predicting mental health problems among migrant workers. Subjective SES reflects the perception and relative position of income and social class via comparison with their social network members. This can therefore be a good alternative index for identifying those with a high risk of mental health problems [39]. Second, reducing interpersonal discrimination toward migrants can alleviate mental health problems among migrant workers. Providing equal access to social welfare and improving the human environment would benefit migrant workers. Specifically, policies and strategies that promote access to education, equal work opportunities, and other areas of social welfare could reduce discrimination [22]. Finally, more attention should be paid to older migrant workers since they are especially prone to interpersonal discrimination and mental health issues, particularly those with low subjective SES.

## Strengths and limitations

This study has some limitations. First, because the data came from a cross-sectional survey, causal relationships could not be established. Second, objective SES was measured only on the basis of monthly income. This was largely because occupation classification is not a sensitive index for reflecting objective income in China. The absence of other common indictors, such as education and occupation, might have limited variations among the migratory population. Finally, although this study was based on a large representative sample from various parts of China, the results might not be applicable to migrants in other countries or regions.

Despite these limitations, this study compared the effects of objective and subjective SES on migrant workers’ mental health and explored some potential mechanisms underlying the relationship between SES and mental health. The findings in the current study can suggest some directions for future research. First, the mediation role of perceived interpersonal discrimination should be examined in the future research on migrants’ mental health. Although China’s dual hukou system has been gradually relaxed, other forms of hukou-based discrimination still exist [73,74]. Second, future studies can test the potential moderation effects of other demographic variables, such as gender, occupation, and education, on the association between SES and mental health among migrant workers to identify vulnerable groups at high risk of mental health problems.

## References

[1] Liang Z., Li Z. & Ma Z. (2014) Changing Patterns of the Floating Population in China during 2000–2010. Population and development review. 40(4), 695–716. doi: 10.1111/j.1728-4457.2014.00007.x 26213427 PMC4508877

[2] National Health and Family Planning Commission of PRC. (2018) *Report On China’s Migrant Population Development*. (China Population Publishing House).

[3] Lu Y. & Wang F. (2013) From general discrimination to segmented inequality: Migration and inequality in urban China. Social Science Research. 42(6), 1443–1456. doi: 10.1016/j.ssresearch.2013.06.006 24090844

[4] Dong X. & Bowles P. (2002) Segmentation and discrimination in China’s emerging industrial labor market. China Economic Review. 13(2), 170–196.

[5] Li X., Stanton B., Fang X. & Lin D. (2006) Social stigma and mental health among rural-to-urban migrants in China: A conceptual framework and future research needs. World health & population. 8(3), 14–31. doi: 10.12927/whp.2006.18282 18277106 PMC2249560

[6] Gong P. et al (2012). Urbanization and health in China. Lancet. 379(9818), 843–852. 22386037 10.1016/S0140-6736(11)61878-3 PMC3733467

[7] Zhong H., Xu J. & Piquero A.R. (2017) Internal Migration, Social Exclusion, and Victimization: An Analysis of Chinese Rural-to-Urban Migrants. Journal of Research in Crime and Delinquency. 54(4), 479–514.

[8] Lin Y. et al (2016) Association between Social Integration and Health among Internal Migrants in Zhongshan, China. PLOS ONE. 11(2), e0148397. doi: 10.1371/journal.pone.0148397 26863008 PMC4749174

[9] Lin D. et al (2011) Discrimination, Perceived Social Inequity, and Mental Health Among Rural-to-Urban Migrants in China. Community Mental Health Journal. 47(2), 171–180. doi: 10.1007/s10597-009-9278-4 20033772 PMC2891847

[10] Wang Y. et al (2018) An analysis of mental health status of female migrant workers in a city. Chinese journal of industrial hygiene and occupational diseases. 36(2), 110–114. doi: 10.3760/cma.j.issn.1001-9391.2018.02.008 29699009

[11] Zhong B. et al (2018) Common mental health problems in rural-to-urban migrant workers in Shenzhen, China: prevalence and risk factors. Epidemiology and Psychiatric Sciences. 27(3), 256–265. doi: 10.1017/S2045796016001141 28067189 PMC6998856

[12] American Psychological Association (2015) Measuring socioeconomic status and subjective social status https://www.apa.org/pi/ses/resources/class/measuring-status/.

[13] Anderson C., Kraus M.W., Galinsky A.D. & Keltner D (2012) The Local-Ladder Effect: Social Status and Subjective Well-Being. Psychological Science. 23(7), 764–771. doi: 10.1177/0956797611434537 22653798

[14] Rarick J.R.D., Dolan C.T., Han W. & Wen J (2018) Relations Between Socioeconomic Status, Subjective Social Status, and Health in Shanghai, China. Social Science Quarterly. 99(1), 390–405.

[15] Gong F., Xu J. & Takeuchi D.T (2012) Beyond conventional socioeconomic status: examining subjective and objective social status with self-reported health among Asian immigrants. Journal of Behavioral Medicine. 35(4), 407–419. doi: 10.1007/s10865-011-9367-z 21720827

[16] Curhan K.B. et al (2014) Subjective and Objective Hierarchies and Their Relations to Psychological Well-Being: A U.S./Japan Comparison. Social Psychological and Personality Science. 5(8), 855–864. 25530829 10.1177/1948550614538461 PMC4266948

[17] Singh-Manoux A., Marmot M.G. & Adler N.E (2005) Does subjective social status predict health and change in health status better than objective status? Psychosomatic medicine. 67(6), 855–861. doi: 10.1097/01.psy.0000188434.52941.a0 16314589

[18] Hebl M.R., Foster J.B., Mannix L.M & Dovidio J.F (2002) Formal and Interpersonal Discrimination: A Field Study of Bias Toward Homosexual Applicants. Personality and Social Psychology Bulletin. 28(6), 815–825.

[19] Fan C.C (2002) The Elite, the Natives, and the Outsiders: Migration and Labor Market Segmentation in Urban China. Annals of the Association of American Geographers. 92(1), 103–124.

[20] Kessler R.C., Mickelson K.D. & Williams D.R (1999) The prevalence, distribution, and mental health correlates of perceived discrimination in the United States. Journal of Health and Social Behavior. 40, 208–230. 10513145

[21] Fuller-Rowell T.E. & Evans G.W. & Ong A.D (2012) Poverty and Health: The Mediating Role of Perceived Discrimination. Psychological Science. 23(7), 734–739. doi: 10.1177/0956797612439720 22700331

[22] Chen X. et.al (2011) Social Stigma, Social Capital Reconstruction and Rural Migrants in Urban China: A Population Health Perspective. Human organization. 70(1), 22–32. doi: 10.17730/humo.70.1.k76047734m703500 21516266 PMC3080703

[23] De Maio F.G. & Kemp E (2010) The deterioration of health status among immigrants to Canada. Global Public Health. 5(5), 462–478. doi: 10.1080/17441690902942480 19513909

[24] Chou K.L. (2012) Perceived discrimination and depression among new migrants to Hong Kong: The moderating role of social support and neighborhood collective efficacy. Journal of Affective Disorders. 138(1), 63–70. 22284018 10.1016/j.jad.2011.12.029

[25] Liu X. & Zhao J. (2016) Chinese Migrant Adolescents’ Perceived Discrimination and Psychological Well-Being: The Moderating Roles of Group Identity and the Type of School. PLOS ONE. 11(1), e0146559. doi: 10.1371/journal.pone.0146559 26731529 PMC4701425

[26] Cheng Z., Chen J. & Wang M. (2014) Urban China in the new era: Market reforms, current state, and the road forward. Springer, Berlin, pp. 125–153.

[27] Zhong B et al. (2017) Mental health of the old- and new-generation migrant workers in China: who are at greater risk for psychological distress?. Oncotarget. 8(35), 59791–99. doi: 10.18632/oncotarget.15985 28938682 PMC5601778

[28] Foner N. (1984) Ages in conflict: A cross-cultural perspective on inequality between old and young. Columbia University Press, New York.

[29] Pampel F.C.(1998) *Aging*, *Social Inequality*, *And Public Policy*. (Sage Publications).

[30] Garstka T.A., Schmitt M.T., Branscombe N.R. & Hummert M.L. (2004) How Young and Older Adults Differ in Their Responses to Perceived Age Discrimination. Psychol Aging. 19(2), 326–335. doi: 10.1037/0882-7974.19.2.326 15222826

[31] Wang D. & He S. *Mobility*, *Sociability And Well-being Of Urban Living*. (Springer, 2016).

[32] Carstensen L.L. (1992) Motivation for social contact across the life span: A theory of socioemotional selectivity. Nebraska Symposium on Motivation. 40, 209–254. 1340521

[33] Blanchard-Fields F., Chen Y. & Norris L. (1997) Everyday problem solving across the adult life span: Influence of domain specificity and cognitive appraisal. Psychology and Aging. 12(4), 684–693. 9416636

[34] Blanchard-Fields F., Stein R. & Watson T.L. (2004) Age differences in emotion-regulation strategies in handling everyday problems. The Journals of Gerontology Series B. 59(6), 261–269. doi: 10.1093/geronb/59.6.p261 15576853

[35] Nolen-Hoeksema S. & Aldao A. (2011) Gender and age differences in emotion regulation strategies and their relationship to depressive symptoms. Personality and Individual Differences. 51(6), 704–708.

[36] Nolen-Hoeksema S., Wisco B.E. & Lyubomirsky S. (2008) Rethinking Rumination. Perspectives on Psychological Science. 3(5), 400–424. 26158958 10.1111/j.1745-6924.2008.00088.x

[37] Aldao A., Nolen-Hoeksema S. & Schweizer S. (2010) Emotion-regulation strategies across psychopathology: A meta-analytic review. Clinical Psychology Review. 30(2), 217–237. doi: 10.1016/j.cpr.2009.11.004 20015584

[38] Johnson S.E., Richeson J.A. & Finkel E.J. (2011) Middle class and marginal? Socioeconomic status, stigma, and self-regulation at an elite university. Journal of personality and social psychology. 100(5), 838. doi: 10.1037/a0021956 21280968

[39] Huang S. et al. (2017) The Effects of Objective and Subjective Socioeconomic Status on Subjective Well-Being among Rural-to-Urban Migrants in China: The Moderating Role of Subjective Social Mobility. Frontiers in Psychology. 8, 819. doi: 10.3389/fpsyg.2017.00819 28588531 PMC5439243

[40] Adler N.E., Epel E.S., Castellazzo G. & lckovics J.R. (2000) Relationship of subjective and objective social status with psychological and physiological functioning: Preliminary data in healthy, White women. Health Psychology. 19(6), 586–592. doi: 10.1037//0278-6133.19.6.586 11129362

[41] Jin L. (2016) Migration, relative deprivation, and psychological well-being in China. American Behavioral Scientist. 60(5–6), 750–770.

[42] Kessler R.C. et al. (2002) Short screening scales to monitor population prevalence and trends in non-specific psychological distress. Psychological medicine. 32(6), 959–976. 12214795 10.1017/s0033291702006074

[43] Kessler R. C. et al. Screening for Serious Mental Illness in the General Population. Arch Gen Psychiatry. 60(2), 184–189 (2003). doi: 10.1001/archpsyc.60.2.184 12578436

[44] Kessler R.C. et al. (2010) Screening for serious mental illness in the general population with the K6 screening scale: results from the WHO World Mental Health (WMH) survey initiative. International journal of methods in psychiatric research. 19(Suppl 1), 4–22. doi: 10.1002/mpr.310 20527002 PMC3659799

[45] Bessaha M.L. (2015) Factor Structure of the Kessler Psychological Distress Scale (K6) Among Emerging Adults. Research on Social Work Practice. 27(5), 616–624.

[46] Green J.G., Gruber M.J., Sampson N.A., Zaslavsky A.M. & Kessler R.C. (2010) Improving the K6 short scale to predict serious emotional disturbance in adolescents in the USA. International journal of methods in psychiatric research. 19(Suppl 1), 23–35. doi: 10.1002/mpr.314 20527003 PMC3686478

[47] Chan S.M. & Fung T.C.T. (2014) Reliability and validity of K10 and K6 in screening depressive symptoms in Hong Kong adolescents. Vulnerable Children and Youth Studies. 9(1), 75–85.

[48] Peiper N., Lee A., Lindsay S., Drashner N. & Wing J. (2016) The performance of the K6 scale in a large school sample: A follow-up study evaluating measurement invariance on the Idaho Youth Prevention Survey. Psychological assessment. 28, 775–779. 10.1037/pas0000188 26214014

[49] United Nations. Who Are the Youth?. https://www.un.org/en/sections/issues-depth/youth-0/index.html/ (accessed 10 Dec 2020)

[50] R Core Team. *R*: *A*. (2019) *Language And Environment For Statistical Computing*. (R Foundation for Statistical Computing).

[51] Hair J.F. (2016) *Multivariate Data Analysis*, 6^th^ ed. (Pearson Prentice Hall).

[52] Curran P.J., West S.G. & Finch J.F. (1996).The robustness of test statistics to nonnormality and specification error in confirmatory factor analysis. Psychological Methods. 1(1), 16–29.

[53] Preacher K.J., Rucker D.D. & Hayes A.F. (2007) Addressing Moderated Mediation Hypotheses: Theory, Methods, and Prescriptions. Multivariate Behavioral Research. 42(1), 185–227. doi: 10.1080/00273170701341316 26821081

[54] Hayes A.F. (2009) Beyond Baron and Kenny: Statistical Mediation Analysis in the New Millennium. Communication Monographs. 76(4), 408–420.

[55] Joreskog K.G. & Sorbom D. (1989) *LISREL 7*: *A Guide To The Program And Applications*. (Spss Inc).

[56] Bentler P.M. (1990) Comparative fit indexes in structural models. Psychological Bulletin. 107(2), 238–246. doi: 10.1037/0033-2909.107.2.238 2320703

[57] Hu L.T., Bentler P.M. (1999) Cutoff criteria for fit indexes in covariance structure analysis: Conventional criteria versus new alternatives. Structural Equation Modeling. 6(1), 1–55.

[58] Fornell C. & Larcker D.F. (1981) Evaluating Structural Equation Models with Unobservable Variables and Measurement Error. Journal of Marketing Research. 18(1), 39–50.

[59] Walker E.R., McGee R.E. & Druss B.G. (2015) Mortality in Mental Disorders and Global Disease Burden Implications: A Systematic Review and Meta-analysis. JAMA Psychiatry. 72(4), 334–341. doi: 10.1001/jamapsychiatry.2014.2502 25671328 PMC4461039

[60] Vigo D., Thornicroft G. & Atun R. (2016) Estimating the true global burden of mental illness. The Lancet Psychiatry. 3(2), 171–178. doi: 10.1016/S2215-0366(15)00505-2 26851330

[61] Schnittker J. & McLeod J.D. (2005) The social psychology of health disparities. Annual Review of Sociology. 31, 75–103.

[62] Kim M.T., Han H.R., Shin H.S., Kim K.B. & Lee H.B. (2005) Factors Associated with Depression Experience of Immigrant Populations: A Study of Korean Immigrants. Archives of Psychiatric Nursing. 19(5), 217–225. doi: 10.1016/j.apnu.2005.07.004 16226673

[63] Kuruvilla A. & Jacob K.S. (2007) Poverty, social stress & mental health. Indian Journal of Medical Research. 126(4), 273. 18032802

[64] Lu, M. & Xia, Y. (2016) Migration in the People’s Republic of China. Asian Development Bank Institute. ADBI Working Paper Series, issue 593. https://www.adb.org/sites/default/files/publication/191876/adbi-wp593.pdf.

[65] Mohabir N., Jiang Y. & Ma R. (2017) Chinese floating migrants: Rural-urban migrant laborer’s intentions to stay or return. Habitat International. 60, 101–110.

[66] Festinger L. (1954).A theory of social comparison processes. Human relations. 7(2), 117–140.

[67] World Health Organization. (2009) Promoting mental health: concepts, emerging evidence, practice: a report of the World Health Organization. https://www.who.int/mental_health/evidence/en/promoting_mhh.pdf.

[68] Crosby F. (1976) A model of egoistical relative deprivation. Psychological Review. 83(2), 85–113.

[69] Meng X. & Zhang J. (2001) The Two-Tier Labor Market in Urban China: Occupational Segregation and Wage Differentials between Urban Residents and Rural Migrants in Shanghai. Journal of Comparative Economics. 29(3), 485–504.

[70] Lee L. (2012) Decomposing wage differentials between migrant workers and urban workers in urban China’s labor markets. China Economic Review. 23(2), 461–470.

[71] Han D. (2010) Policing and racialization of rural migrant workers in Chinese cities. Ethnic and Racial Studies. 33(4), 593–610.

[72] Watson A. (2009) Social security for China’s migrant workers-providing for old age. Journal of Current Chinese Affairs. 38(4), 85–115.

[73] Zheng S., Long F., Fan C.C. & Gu Y. (2009) Urban villages in China: A 2008 survey of migrant settlements in Beijing. Eurasian Geography and Economics. 50(4), 425–446.

[74] Tao L., Hui E.C.M., Wong K.W. & Chen T. (2015) Housing choices of migrant workers in China: Beyond the Hukou perspective. Habitat International. 49, 474–483.

